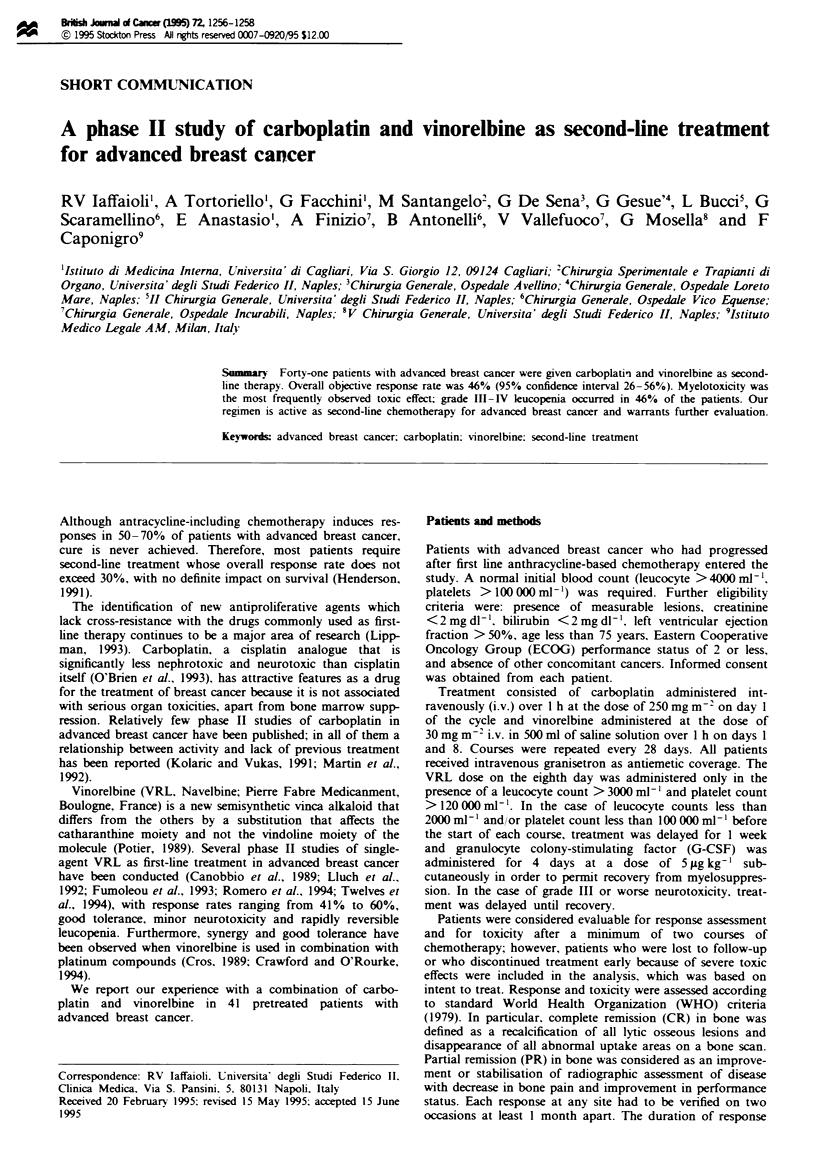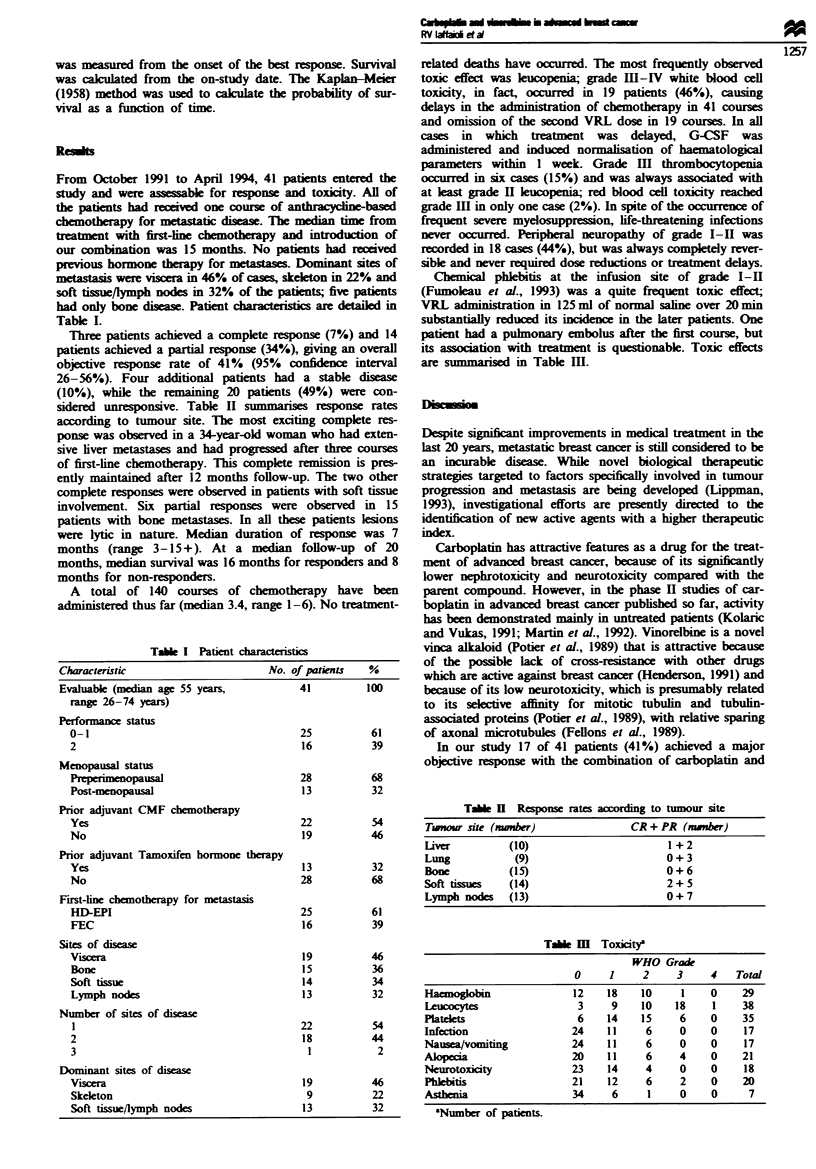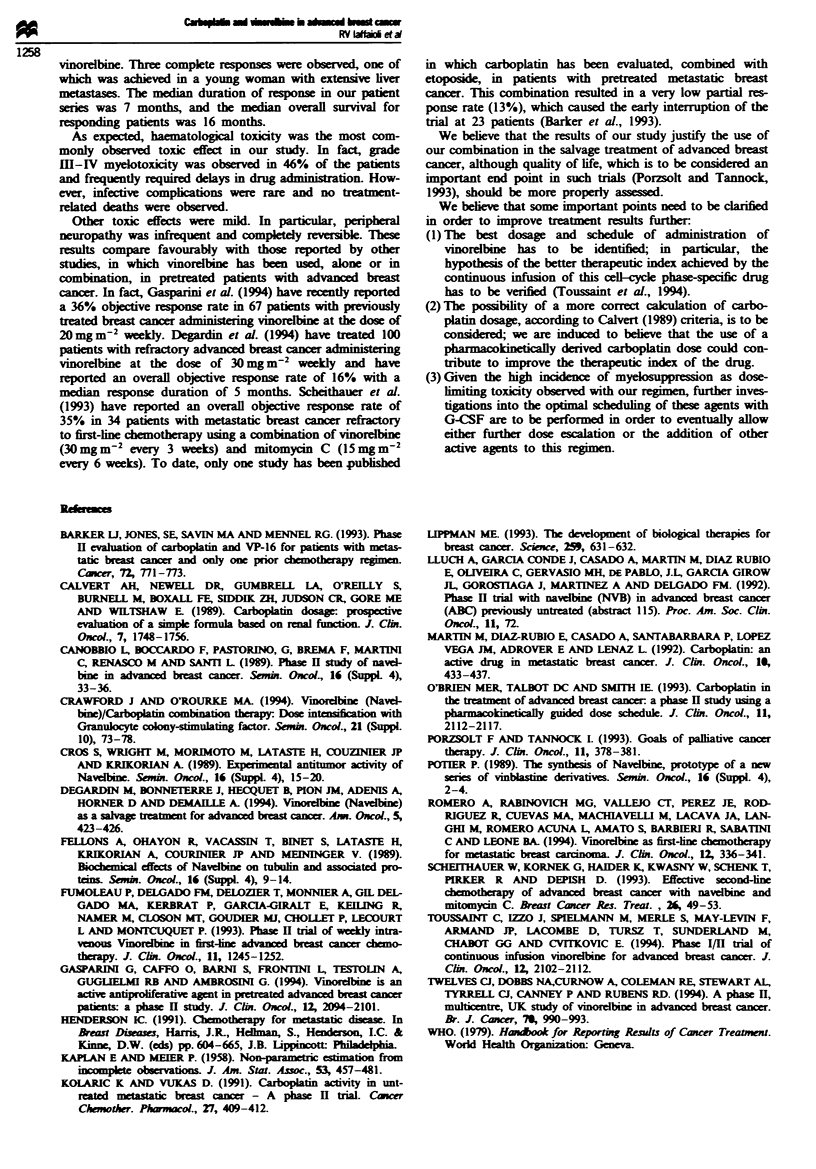# A phase II study of carboplatin and vinorelbine as second-line treatment for advanced breast cancer.

**DOI:** 10.1038/bjc.1995.496

**Published:** 1995-11

**Authors:** R. V. Iaffaioli, A. Tortoriello, G. Facchini, M. Santangelo, G. De Sena, G. Gesue, L. Bucci, G. Scaramellino, E. Anastasio, A. Finizio

**Affiliations:** Istituto di Medicina Interna, Università di Cagliari, Italy.

## Abstract

Forty-one patients with advanced breast cancer were given carboplatin and vinorelbine as second-line therapy. Overall objective response rate was 46% (95% confidence interval 26-56%). Myelotoxicity was the most frequently observed toxic effect; grade III-IV leucopenia occurred in 46% of the patients. Our regimen is active as second-line chemotherapy for advanced breast cancer and warrants further evaluation.


British Journal of Cancer (1995) 72, 1256-1258

© 1995 Stockton Press All rights reserved 0007-0920/95 $12.00

SHORT COMMUNICATION

### A phase II study of carboplatin and vinorelbine as second-line treatment for advanced breast cancer

RV Iaffaioli[1], A Tortoriello[1], G Facchini[1], M Santangelo[2], G De Sena[3], G Gesue'[4], L Bucci[5], G Scaramellino[6], E Anastasio[1], A Finizio[7], B Antonelli[6], V Vallefuoco[7], G Mosella[8] and F Caponigro[9]

[1]*Istituto di Medicina Interna, Universita' di Cagliari, Via S. Giorgio 12, 09124 Cagliari;* [2]*Chirurgia Sperimentale e Trapianti di Organo, Universita' degli Studi Federico II, Naples;* [3]*Chirurgia Generale, Ospedale Avellino;* [4]*Chirurgia Generale, Ospedale Loreto Mare, Naples;* [5]*II Chirurgia Generale, Universita' degli Studi Federico II, Naples;* [6]*Chirurgia Generale, Ospedale Vico Equense;* [7]*Chirurgia Generale, Ospedale Incurabili, Naples;* [8]*V Chirurgia Generale, Universita' degli Studi Federico II, Naples;* [9]*Istituto Medico Legale AM, Milan, Italy*

**Summary** Forty-one patients with advanced breast cancer were given carboplatin and vinorelbine as second-line therapy. Overall objective response rate was 46% (95% confidence interval 26-56%). Myelotoxicity was the most frequently observed toxic effect; grade III-IV leucopenia occurred in 46% of the patients. Our regimen is active as second-line chemotherapy for advanced breast cancer and warrants further evaluation.

**Keywords:** advanced breast cancer; carboplatin; vinorelbine; second-line treatment

Although antracycline-including chemotherapy induces responses in 50-70% of patients with advanced breast cancer, cure is never achieved. Therefore, most patients require second-line treatment whose overall response rate does not exceed 30%, with no definite impact on survival (Henderson, 1991).

The identification of new antiproliferative agents which lack cross-resistance with the drugs commonly used as first-line therapy continues to be a major area of research (Lippman, 1993). Carboplatin, a cisplatin analogue that is significantly less nephrotoxic and neurotoxic than cisplatin itself (O'Brien *et al.*, 1993), has attractive features as a drug for the treatment of breast cancer because it is not associated with serious organ toxicities, apart from bone marrow suppression. Relatively few phase II studies of carboplatin in advanced breast cancer have been published; in all of them a relationship between activity and lack of previous treatment has been reported (Kolaric and Vukas, 1991; Martin *et al.*, 1992).

Vinorelbine (VRL, Navelbine; Pierre Fabre Medicanment, Boulogne, France) is a new semisynthetic vinca alkaloid that differs from the others by a substitution that affects the catharanthine moiety and not the vindoline moiety of the molecule (Potier, 1989). Several phase II studies of single-agent VRL as first-line treatment in advanced breast cancer have been conducted (Canobbio *et al.*, 1989; Lluch *et al.*, 1992; Fumoleou *et al.*, 1993; Romero *et al.*, 1994; Twelves *et al.*, 1994), with response rates ranging from 41% to 60%, good tolerance, minor neurotoxicity and rapidly reversible leucopenia. Furthermore, synergy and good tolerance have been observed when vinorelbine is used in combination with platinum compounds (Cros, 1989; Crawford and O'Rourke, 1994).

We report our experience with a combination of carboplatin and vinorelbine in 41 pretreated patients with advanced breast cancer.

Correspondence: RV Iaffaioli, Universita' degli Studi Federico II, Clinica Medica, Via S. Pansini, 5, 80131 Napoli, Italy
Received 20 February 1995; revised 15 May 1995; accepted 15 June 1995

### Patients and methods

Patients with advanced breast cancer who had progressed after first line anthracycline-based chemotherapy entered the study. A normal initial blood count (leucocyte $>4000\ ml^{-1}$, platelets $>100\,000\ ml^{-1}$) was required. Further eligibility criteria were: presence of measurable lesions, creatinine $<2\ mg\ dl^{-1}$, bilirubin $<2\ mg\ dl^{-1}$, left ventricular ejection fraction $>50\%$, age less than 75 years, Eastern Cooperative Oncology Group (ECOG) performance status of 2 or less, and absence of other concomitant cancers. Informed consent was obtained from each patient.

Treatment consisted of carboplatin administered intravenously (i.v.) over 1 h at the dose of $250\ mg\ m^{-2}$ on day 1 of the cycle and vinorelbine administered at the dose of $30\ mg\ m^{-2}$ i.v. in 500 ml of saline solution over 1 h on days 1 and 8. Courses were repeated every 28 days. All patients received intravenous granisetron as antiemetic coverage. The VRL dose on the eighth day was administered only in the presence of a leucocyte count $>3000\ ml^{-1}$ and platelet count $>120\,000\ ml^{-1}$. In the case of leucocyte counts less than $2000\ ml^{-1}$ and/or platelet count less than $100\,000\ ml^{-1}$ before the start of each course, treatment was delayed for 1 week and granulocyte colony-stimulating factor (G-CSF) was administered for 4 days at a dose of $5\ \mu g\ kg^{-1}$ subcutaneously in order to permit recovery from myelosuppression. In the case of grade III or worse neurotoxicity, treatment was delayed until recovery.

Patients were considered evaluable for response assessment and for toxicity after a minimum of two courses of chemotherapy; however, patients who were lost to follow-up or who discontinued treatment early because of severe toxic effects were included in the analysis, which was based on intent to treat. Response and toxicity were assessed according to standard World Health Organization (WHO) criteria (1979). In particular, complete remission (CR) in bone was defined as a recalcification of all lytic osseous lesions and disappearance of all abnormal uptake areas on a bone scan. Partial remission (PR) in bone was considered as an improvement or stabilisation of radiographic assessment of disease with decrease in bone pain and improvement in performance status. Each response at any site had to be verified on two occasions at least 1 month apart. The duration of response was measured from the onset of the best response. Survival was calculated from the on-study date. The Kaplan–Meier (1958) method was used to calculate the probability of survival as a function of time.

### Results

From October 1991 to April 1994, 41 patients entered the study and were assessable for response and toxicity. All of the patients had received one course of anthracycline-based chemotherapy for metastatic disease. The median time from treatment with first-line chemotherapy and introduction of our combination was 15 months. No patients had received previous hormone therapy for metastases. Dominant sites of metastasis were viscera in 46% of cases, skeleton in 22% and soft tissue/lymph nodes in 32% of the patients; five patients had only bone disease. Patient characteristics are detailed in Table I.

Three patients achieved a complete response (7%) and 14 patients achieved a partial response (34%), giving an overall objective response rate of 41% (95% confidence interval 26–56%). Four additional patients had a stable disease (10%), while the remaining 20 patients (49%) were considered unresponsive. Table II summarises response rates according to tumour site. The most exciting complete response was observed in a 34-year-old woman who had extensive liver metastases and had progressed after three courses of first-line chemotherapy. This complete remission is presently maintained after 12 months follow-up. The two other complete responses were observed in patients with soft tissue involvement. Six partial responses were observed in 15 patients with bone metastases. In all these patients lesions were lytic in nature. Median duration of response was 7 months (range 3–15+). At a median follow-up of 20 months, median survival was 16 months for responders and 8 months for non-responders.

A total of 140 courses of chemotherapy have been administered thus far (median 3.4, range 1–6). No treatment-related deaths have occurred. The most frequently observed toxic effect was leucopenia; grade III–IV white blood cell toxicity, in fact, occurred in 19 patients (46%), causing delays in the administration of chemotherapy in 41 courses and omission of the second VRL dose in 19 courses. In all cases in which treatment was delayed, G-CSF was administered and induced normalisation of haematological parameters within 1 week. Grade III thrombocytopenia occurred in six cases (15%) and was always associated with at least grade II leucopenia; red blood cell toxicity reached grade III in only one case (2%). In spite of the occurrence of frequent severe myelosuppression, life-threatening infections never occurred. Peripheral neuropathy of grade I–II was recorded in 18 cases (44%), but was always completely reversible and never required dose reductions or treatment delays.

Chemical phlebitis at the infusion site of grade I–II (Fumoleau *et al.*, 1993) was a quite frequent toxic effect; VRL administration in 125 ml of normal saline over 20 min substantially reduced its incidence in the later patients. One patient had a pulmonary embolus after the first course, but its association with treatment is questionable. Toxic effects are summarised in Table III.

**Table I** Patient characteristics

| *Characteristic* | *No. of patients* | *%* |
|---|---|---|
| Evaluable (median age 55 years, range 26–74 years) | 41 | 100 |
| Performance status | | |
| 0–1 | 25 | 61 |
| 2 | 16 | 39 |
| Menopausal status | | |
| Preperimenopausal | 28 | 68 |
| Post-menopausal | 13 | 32 |
| Prior adjuvant CMF chemotherapy | | |
| Yes | 22 | 54 |
| No | 19 | 46 |
| Prior adjuvant Tamoxifen hormone therapy | | |
| Yes | 13 | 32 |
| No | 28 | 68 |
| First-line chemotherapy for metastasis | | |
| HD-EPI | 25 | 61 |
| FEC | 16 | 39 |
| Sites of disease | | |
| Viscera | 19 | 46 |
| Bone | 15 | 36 |
| Soft tissue | 14 | 34 |
| Lymph nodes | 13 | 32 |
| Number of sites of disease | | |
| 1 | 22 | 54 |
| 2 | 18 | 44 |
| 3 | 1 | 2 |
| Dominant sites of disease | | |
| Viscera | 19 | 46 |
| Skeleton | 9 | 22 |
| Soft tissue/lymph nodes | 13 | 32 |

### Discussion

Despite significant improvements in medical treatment in the last 20 years, metastatic breast cancer is still considered to be an incurable disease. While novel biological therapeutic strategies targeted to factors specifically involved in tumour progression and metastasis are being developed (Lippman, 1993), investigational efforts are presently directed to the identification of new active agents with a higher therapeutic index.

Carboplatin has attractive features as a drug for the treatment of advanced breast cancer, because of its significantly lower nephrotoxicity and neurotoxicity compared with the parent compound. However, in the phase II studies of carboplatin in advanced breast cancer published so far, activity has been demonstrated mainly in untreated patients (Kolaric and Vukas, 1991; Martin *et al.*, 1992). Vinorelbine is a novel vinca alkaloid (Potier *et al.*, 1989) that is attractive because of the possible lack of cross-resistance with other drugs which are active against breast cancer (Henderson, 1991) and because of its low neurotoxicity, which is presumably related to its selective affinity for mitotic tubulin and tubulin-associated proteins (Potier *et al.*, 1989), with relative sparing of axonal microtubules (Fellons *et al.*, 1989).

In our study 17 of 41 patients (41%) achieved a major objective response with the combination of carboplatin and

**Table II** Response rates according to tumour site

| *Tumour site* | *(number)* | *CR + PR (number)* |
|---|---|---|
| Liver | (10) | 1 + 2 |
| Lung | (9) | 0 + 3 |
| Bone | (15) | 0 + 6 |
| Soft tissues | (14) | 2 + 5 |
| Lymph nodes | (13) | 0 + 7 |

**Table III** Toxicity[a]

| | *WHO Grade* | | | | | |
|---|---|---|---|---|---|---|
| | *0* | *1* | *2* | *3* | *4* | *Total* |
| Haemoglobin | 12 | 18 | 10 | 1 | 0 | 29 |
| Leucocytes | 3 | 9 | 10 | 18 | 1 | 38 |
| Platelets | 6 | 14 | 15 | 6 | 0 | 35 |
| Infection | 24 | 11 | 6 | 0 | 0 | 17 |
| Nausea/vomiting | 24 | 11 | 6 | 0 | 0 | 17 |
| Alopecia | 20 | 11 | 6 | 4 | 0 | 21 |
| Neurotoxicity | 23 | 14 | 4 | 0 | 0 | 18 |
| Phlebitis | 21 | 12 | 6 | 2 | 0 | 20 |
| Asthenia | 34 | 6 | 1 | 0 | 0 | 7 |

[a]Number of patients.

vinorelbine. Three complete responses were observed, one of which was achieved in a young woman with extensive liver metastases. The median duration of response in our patient series was 7 months, and the median overall survival for responding patients was 16 months.

As expected, haematological toxicity was the most commonly observed toxic effect in our study. In fact, grade III–IV myelotoxicity was observed in 46% of the patients and frequently required delays in drug administration. However, infective complications were rare and no treatment-related deaths were observed.

Other toxic effects were mild. In particular, peripheral neuropathy was infrequent and completely reversible. These results compare favourably with those reported by other studies, in which vinorelbine has been used, alone or in combination, in pretreated patients with advanced breast cancer. In fact, Gasparini *et al.* (1994) have recently reported a 36% objective response rate in 67 patients with previously treated breast cancer administering vinorelbine at the dose of $20 \, mg \, m^{-2}$ weekly. Degardin *et al.* (1994) have treated 100 patients with refractory advanced breast cancer administering vinorelbine at the dose of $30 \, mg \, m^{-2}$ weekly and have reported an overall objective response rate of 16% with a median response duration of 5 months. Scheithauer *et al.* (1993) have reported an overall objective response rate of 35% in 34 patients with metastatic breast cancer refractory to first-line chemotherapy using a combination of vinorelbine ($30 \, mg \, m^{-2}$ every 3 weeks) and mitomycin C ($15 \, mg \, m^{-2}$ every 6 weeks). To date, only one study has been published in which carboplatin has been evaluated, combined with etoposide, in patients with pretreated metastatic breast cancer. This combination resulted in a very low partial response rate (13%), which caused the early interruption of the trial at 23 patients (Barker *et al.*, 1993).

We believe that the results of our study justify the use of our combination in the salvage treatment of advanced breast cancer, although quality of life, which is to be considered an important end point in such trials (Porzsolt and Tannock, 1993), should be more properly assessed.

We believe that some important points need to be clarified in order to improve treatment results further:

(1) The best dosage and schedule of administration of vinorelbine has to be identified; in particular, the hypothesis of the better therapeutic index achieved by the continuous infusion of this cell–cycle phase-specific drug has to be verified (Toussaint *et al.*, 1994).
(2) The possibility of a more correct calculation of carboplatin dosage, according to Calvert (1989) criteria, is to be considered; we are induced to believe that the use of a pharmacokinetically derived carboplatin dose could contribute to improve the therapeutic index of the drug.
(3) Given the high incidence of myelosuppression as dose-limiting toxicity observed with our regimen, further investigations into the optimal scheduling of these agents with G-CSF are to be performed in order to eventually allow either further dose escalation or the addition of other active agents to this regimen.

### References

BARKER LJ, JONES, SE, SAVIN MA AND MENNEL RG. (1993). Phase II evaluation of carboplatin and VP-16 for patients with metastatic breast cancer and only one prior chemotherapy regimen. *Cancer*, **72**, 771–773.

CALVERT AH, NEWELL DR, GUMBRELL LA, O'REILLY S, BURNELL M, BOXALL FE, SIDDIK ZH, JUDSON CR, GORE ME AND WILTSHAW E. (1989). Carboplatin dosage: prospective evaluation of a simple formula based on renal function. *J. Clin. Oncol.*, **7**, 1748–1756.

CANOBBIO L, BOCCARDO F, PASTORINO, G, BREMA F, MARTINI C, RENASCO M AND SANTI L. (1989). Phase II study of navelbine in advanced breast cancer. *Semin. Oncol.*, **16** (Suppl. 4), 33–36.

CRAWFORD J AND O'ROURKE MA. (1994). Vinorelbine (Navelbine)/Carboplatin combination therapy: Dose intensification with Granulocyte colony-stimulating factor. *Semin. Oncol.*, **21** (Suppl. 10), 73–78.

CROS S, WRIGHT M, MORIMOTO M, LATASTE H, COUZINIER JP AND KRIKORIAN A. (1989). Experimental antitumor activity of Navelbine. *Semin. Oncol.*, **16** (Suppl. 4), 15–20.

DEGARDIN M, BONNETERRE J, HECQUET B, PION JM, ADENIS A, HORNER D AND DEMAILLE A. (1994). Vinorelbine (Navelbine) as a salvage treatment for advanced breast cancer. *Ann. Oncol.*, **5**, 423–426.

FELLONS A, OHAYON R, VACASSIN T, BINET S, LATASTE H, KRIKORIAN A, COURINIER JP AND MEININGER V. (1989). Biochemical effects of Navelbine on tubulin and associated proteins. *Semin. Oncol.*, **16** (Suppl. 4), 9–14.

FUMOLEAU P, DELGADO FM, DELOZIER T, MONNIER A, GIL DELGADO MA, KERBRAT P, GARCIA-GIRALT E, KEILING R, NAMER M, CLOSON MT, GOUDIER MJ, CHOLLET P, LECOURT L AND MONTCUQUET P. (1993). Phase II trial of weekly intravenous Vinorelbine in first-line advanced breast cancer chemotherapy. *J. Clin. Oncol.*, **11**, 1245–1252.

GASPARINI G, CAFFO O, BARNI S, FRONTINI L, TESTOLIN A, GUGLIELMI RB AND AMBROSINI G. (1994). Vinorelbine is an active antiproliferative agent in pretreated advanced breast cancer patients: a phase II study. *J. Clin. Oncol.*, **12**, 2094–2101.

HENDERSON IC. (1991). Chemotherapy for metastatic disease. In *Breast Diseases*, Harris, J.R., Hellman, S., Henderson, I.C. & Kinne, D.W. (eds) pp. 604–665, J.B. Lippincott: Philadelphia.

KAPLAN E AND MEIER P. (1958). Non-parametric estimation from incomplete observations. *J. Am. Stat. Assoc.*, **53**, 457–481.

KOLARIC K AND VUKAS D. (1991). Carboplatin activity in untreated metastatic breast cancer – A phase II trial. *Cancer Chemother. Pharmacol.*, **27**, 409–412.

LIPPMAN ME. (1993). The development of biological therapies for breast cancer. *Science*, **259**, 631–632.

LLUCH A, GARCIA CONDE J, CASADO A, MARTIN M, DIAZ RUBIO E, OLIVEIRA C, GERVASIO MH, DE PABLO, J.L, GARCIA GIROW JL, GOROSTIAGA J, MARTINEZ A AND DELGADO FM. (1992). Phase II trial with navelbine (NVB) in advanced breast cancer (ABC) previously untreated (abstract 115). *Proc. Am. Soc. Clin. Oncol.*, **11**, 72.

MARTIN M, DIAZ-RUBIO E, CASADO A, SANTABARBARA P, LOPEZ VEGA JM, ADROVER E AND LENAZ L. (1992). Carboplatin: an active drug in metastatic breast cancer. *J. Clin. Oncol.*, **10**, 433–437.

O'BRIEN MER, TALBOT DC AND SMITH IE. (1993). Carboplatin in the treatment of advanced breast cancer: a phase II study using a pharmacokinetically guided dose schedule. *J. Clin. Oncol.*, **11**, 2112–2117.

PORZSOLT F AND TANNOCK I. (1993). Goals of palliative cancer therapy. *J. Clin. Oncol.*, **11**, 378–381.

POTIER P. (1989). The synthesis of Navelbine, prototype of a new series of vinblastine derivatives. *Semin. Oncol.*, **16** (Suppl. 4), 2–4.

ROMERO A, RABINOVICH MG, VALLEJO CT, PEREZ JE, RODRIGUEZ R, CUEVAS MA, MACHIAVELLI M, LACAVA JA, LANGHI M, ROMERO ACUNA L, AMATO S, BARBIERI R, SABATINI C AND LEONE BA. (1994). Vinorelbine as first-line chemotherapy for metastatic breast carcinoma. *J. Clin. Oncol.*, **12**, 336–341.

SCHEITHAUER W, KORNEK G, HAIDER K, KWASNY W, SCHENK T, PIRKER R AND DEPISH D. (1993). Effective second-line chemotherapy of advanced breast cancer with navelbine and mitomycin C. *Breast Cancer Res. Treat.* , **26**, 49–53.

TOUSSAINT C, IZZO J, SPIELMANN M, MERLE S, MAY-LEVIN F, ARMAND JP, LACOMBE D, TURSZ T, SUNDERLAND M, CHABOT GG AND CVITKOVIC E. (1994). Phase I/II trial of continuous infusion vinorelbine for advanced breast cancer. *J. Clin. Oncol.*, **12**, 2102–2112.

TWELVES CJ, DOBBS NA,CURNOW A, COLEMAN RE, STEWART AL, TYRRELL CJ, CANNEY P AND RUBENS RD. (1994). A phase II, multicentre, UK study of vinorelbine in advanced breast cancer. *Br. J. Cancer*, **70**, 990–993.

WHO. (1979). *Handbook for Reporting Results of Cancer Treatment*. World Health Organization: Geneva.